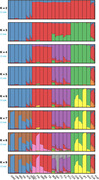# Comprehensive Analysis of Genetic Contributions to Alzheimer’s Disease and Frontotemporal Dementia in Admixed Latin American Populations

**DOI:** 10.1002/alz.093055

**Published:** 2025-01-03

**Authors:** Juliana Acosta‐Uribe, Stefanie Danielle Pina Escudero, J. Nicholas Cochran, Jared W Taylor, Caroline Warly Solsberg, Diana Matallana, Leonel Tadao Takada, Martin Alejandro Bruno, Alexandra R. Levine, Dawwod S. George, Francisco Lopera, Andrea Slachevsky Chonchol, María Isabel Behrens, José Alberto Ávila Funes, Lina Maria Zapata‐Restrepo, Luis Ignacio Brusco, Nilton Custodio, Teresita Ramos Franco, Bárbara Bruna, Daniela P Ponce, Nancy Gelvez, Greizy Lopez, Luisa Gomez, Carlos Felipe Buitrago, Pablo A Reyes, Dafne Estefania Durón, Caroline Pantazis, Marcelo Adrian Maito, Shireen Javandel, Maria Eugenia Godoy, Maria Beatriz Bistue Millon, Dan Vitale, Mike A Nalls, Andrew Singleton, Bruce L. Miller, Agustín Ibáñez, Kenneth S. Kosik, Jennifer S. Yokoyama, Rosa Montesinos, Elisa de Paula França Resende, Multi‐Partner Consortium to Expand Dementia Research in Latin America (ReDLat)

**Affiliations:** ^1^ University of California Santa Barbara, Santa Barbara, CA USA; ^2^ Neurosciences Group of Antioquia, University of Antioquia, Medellin Colombia; ^3^ Global Brain Health Institute (GBHI), University of California, San Francisco USA; ^4^ University of California, San Francisco, San Francisco, CA USA; ^5^ HudsonAlpha Institute for Biotechnology, Huntsville, AL USA; ^6^ University of California San Francisco, San Francisco, CA USA; ^7^ Pontificia Universidad Javeriana, Bogota, Cundinamarca Colombia; ^8^ Hospital Universitario San Ignacio, Bogotá Colombia; ^9^ Hospital Universitario Fundación Santa Fe, Bogotá Colombia; ^10^ Hospital das Clínicas, University of Sao Paulo Medical School, São Paulo Brazil; ^11^ Instituto de Ciencias Biomédicas (ICBM) Facultad de Ciencias Médicas, Universidad Catoóica de Cuyo, San Juan Argentina; ^12^ CONICET, San Juan Argentina; ^13^ University of California, Santa Barbara, Santa Barbara, CA USA; ^14^ Grupo de Neurociencias de Antioquia, Facultad de Medicina, Universidad de Antioquia, Medellín Colombia; ^15^ Neurology Service, Department of Medicine, Clínica Alemana‐Universidad del Desarrollo, Santiago, Chile., Santiago Chile; ^16^ Neuropsychology and Clinical Neuroscience Laboratory (LANNEC), Physiopathology Department ‐ ICBM, Neuroscience and East Neuroscience Departments, Faculty of Medicine, Universidad de Chile, Santiago Chile; ^17^ Geroscience Center for Brain Health and Metabolism (GERO), Santiago Chile; ^18^ Memory and Neuropsychiatric Center (CMYN), Neurology Department, Hospital del Salvador and Faculty of Medicine, Universidad de Chile, Santiago Chile; ^19^ Centro de Investigación Clínica Avanza (CICA), Hospital Clínico Universidad de Chile, Santiago Chile; ^20^ Hospital Clínico de la Universidad de Chile, Santiago de Chile Chile; ^21^ Instituto Nacional de Ciencias Médicas y Nutrición Salvador Zubirán, Mexico City, DF Mexico; ^22^ Facultad de Ciencias de la Salud, Universidad Icesi, Cali, Colombia, Cali Colombia; ^23^ Fundacion Valle del Lili, Cali Colombia; ^24^ ALZAR ‐ Argentine Alzheimer’s Association, Buenos Aires, CABA Argentina; ^25^ Universidad de Buenos Aires, Buenos Aires Argentina; ^26^ Instituto Peruano de Neurociencias, Lima, Lima Peru; ^27^ Hospital del Salvador & Faculty of Medicine, University of Chile., Santiago Chile; ^28^ Pontificia Universidad Javeriana, Bogotá Colombia; ^29^ Pontificia Universidad Javeriana, Bogotá, Cundinamarca Colombia; ^30^ Pontificia Universidad Javeriana, Bogota Colombia; ^31^ Instituto Nacional de Ciencias Médicas y Nutrición Salvador Zubirán, Ciudad de México, DF Mexico; ^32^ Center for Alzheimer’s and Related Dementias, National Institute on Aging and National Institute of Neurological Disorders and Stroke, National Institutes of Health, Bethesda, MD USA; ^33^ Latin American Brain Health Institute (BrainLat), Universidad Adolfo Ibañez, Santiago Chile; ^34^ Universidad de San Andres, Buenos Aires Argentina; ^35^ Universidad Católica de Cuyo / CONICET, San Juan Argentina; ^36^ Instituto de Ciencias Biomédicas (ICBM), Facultad de Ciencias Médicas, Universidad Católica de Cuyo, San Juan Argentina; ^37^ CONICET, San juan Argentina; ^38^ Data Tecnica International, Washington, DC USA; ^39^ National Institute on Aging, National Institutes of Health, Bethesda, MD USA; ^40^ Global Brain Health Institute, San Francisco, CA USA; ^41^ BrainLat Institute, Santiago, Santiago Chile; ^42^ Global Brain Health Institute, Trinity College Dublin, Dublin Ireland; ^43^ Global Brain Health Institute, University of California San Francisco, San Francisco, CA USA; ^44^ Unit Cognitive Impairment and Dementia Prevention, Peruvian Institute of Neurosciences, Lima, Peru, Lima, Lima Peru; ^45^ Research unit, Instituto Peruano de Neurociencias, Lima Peru; ^46^ Faculdade de Medicina de Ciências Médicas de Minas Gerais, Belo Horizonte Brazil; ^47^ Global Brain Health Institute, University of California, San Francisco, CA USA

## Abstract

**Background:**

Most research initiatives have emerged from high‐income countries (HIC), leaving a gap in understanding the disease’s genetic basis in diverse populations like those in Latin American countries (LAC). ReDLat tackles this gap, focusing on LAC’s unique genetics and socioeconomic factors to identify specific Alzheimer’s Disease (AD) and Frontotemporal Dementia (FTD) risk factors in Mexico, Colombia, Peru, Chile, Argentina, and Brazil.

**Method:**

We employed a comprehensive genetic analysis approach, integrating Whole Genome Sequencing (WGS), Exome Sequencing, and SNP arrays to understand the cohort’s unique genetic architecture. We conducted ancestry analysis and searched for disease‐causing variants with mendelian inheritance, genome‐wide association studies (GWAS), rare variant enrichment, and evaluation of Polygenic Risk Scores (PRS).

**Results:**

We recruited and genotyped an initial cohort of 1046 participants with AD, 423 with FTD, and 855 healthy controls (HC) between 2020 and 2023. Analysis is ongoing, and we expect to sequence ∼600 additional samples in the coming months. Ancestry analysis revealed tri‐continental admixture, except for Brazil, which showed an additional Asian component (Figure 1). Top candidate gene rare variant enrichment associations (SKAT p < 0.05) were *TREM2* for FTD and *ABCA7* and *ABCA1* for AD. GWAS identified a robust association with the *APOE* locus on chromosome 19 in AD vs. HC.. We tested an AD PRS developed in European populations by Bellenguez et al (2020). on our cohort using 83 single‐nucleotide polymorphisms.. The PRS modestly distinguishes between all patients and HC (p = 2.4 × 10^‐12), AD vs. HC (p = 2.2 × 10^‐12), and even FTD vs. HC (p = 4.3 × 10^‐5), albeit with modest separation between groups, as expected for its application in a genetically admixed population.

**Conclusion:**

Our findings represent a pivotal step in understanding the genetic landscape of AD and FTD in admixed populations. They underscore the importance of including diverse populations in genetic research, paving the way for future studies. These findings have the potential to inform more personalized approaches to the diagnosis and treatment of neurodegenerative diseases in diverse global populations, as well as identify novel targets for therapeutic development.